# Supplementary material for: The neuromuscular system of Chironomus vitellinus (Diptera: Chironomidae)
Source: PLoS One. 2026 Jan 29;21(1):e0326394. doi: 10.1371/journal.pone.0326394 (PMC12854417; doi:10.1371/journal.pone.0326394)
Supplement: S1 Table — Raw data collected from 19 synapses across 10 animals describing the number of synaptic boutons (Fig 7D) and the dimension of the muscle that is innervated (Fig 7E). (DOCX) [file pone.0326394.s001.docx]

| **NMJ** | **bouton #** | **Area (µm2)** |
| --- | --- | --- |
| 1.1 | 103 | 37564.25 |
| 1.2 | 87 | 43958.94 |
| 2.1 | 113 | 69982.111 |
| 2.2 | 99 | 51705.069 |
| 3.1 | 107 | 23159.515 |
| 3.2 | 86 | 22017.389 |
| 4.1 | 140 | 34251.631 |
| 4.2 | 105 | 37558.183 |
| 5.1 | 101 | 35137.423 |
| 5.2 | 112 | 31850.589 |
| 6.1 | 57 | 45612.216 |
| 6.2 | 66 | 44782.544 |
| 7.1 | 73 | 56974.317 |
| 8.1 | 54 | 61018.018 |
| 8.2 | 47 | 57898.028 |
| 9.1 | 76 | 42542.28 |
| 9.2 | 86 | 34969.062 |
| 10.1 | 67 | 63578.32 |
| 10.2 | 72 | 53347.727 |

**S1 Table**. **Number of synaptic boutons and muscle sizes at the Chironomus VEL 2 NMJ segment A1.** Raw data collected from 19 synapses across 10 animals describing the number of synaptic boutons (Fig 7 D) and the dimension of the muscle that is innervated (Fig 7 E).
