# Supplementary material for: The neuromuscular system of Chironomus vitellinus (Diptera: Chironomidae)
Source: PLoS One. 2026 Jan 29;21(1):e0326394. doi: 10.1371/journal.pone.0326394 (PMC12854417; doi:10.1371/journal.pone.0326394)
Supplement: S2 Table — Raw data collected from 19 synapses across 10 animals describing the number of synaptic boutons. The mean, standard deviation and N number are shown. (DOCX) [file pone.0326394.s002.docx]

| NMJ | Bouton number | |
| --- | --- | --- |
| 1.1 | 62 |  |
| 1.2 | 90 |  |
| 2.1 | 73 |  |
| 2.2 | 82 |  |
| 3.1 | 67 |  |
| 3.2 | 62 |  |
| 4.1 | 69 |  |
| 4.2 | 75 |  |
| 5.1 | 78 |  |
| 5.2 | 99 |  |
| 6.1 | 79 |  |
| 6.2 | 84 |  |
| 7.1 | 80 |  |
| 7.2 | 116 |  |
| 8.1 | 108 |  |
| 8.2 | 89 |  |
| 9.1 | 71 |  |
| 9.2 | 75 |  |
| 10.1 | 48 |  |
|  |  |  |
|  |  |  |
| Mean | 79.31579 |  |
| STDEV | 16.29605 |  |
| N | 19 |  |

**S2 Table**. **Number of synaptic boutons at the Drosophila m6/7 NMJ segment A3**. Raw data collected from 19 synapses across 10 animals describing the number of synaptic boutons. The mean, standard deviation and N number are shown.
